# Identifying Subgroups At-Risk for Noncommunicable Diseases in Cambodia: A Latent Class Analysis of Behavioral and Metabolic Risk Factor Patterns

**DOI:** 10.1007/s44197-025-00464-0

**Published:** 2025-10-13

**Authors:** Cassandra Comey, Kanya Anindya, Ailiana Santosa, Paul Kowal, Srean Chhim, Heng Sopheab, Nawi Ng

**Affiliations:** 1 School of Public Health and Community Medicine, Sahlgrenska Academy University of Gothenburg, Gothenburg, Sweden; 2 Health Data Analytics Team, National Centre for Epidemiology and Population Health, The Australian National University, Canberra, Australia; 3 School of Public Health, National Institute of Public Health, Phnom Penh, Cambodia; 4 Julius Center for Health Sciences and Primary Care, University Medical Center Utrecht, Utrecht, The Netherlands

**Keywords:** Noncommunicable diseases, Risk factors, Latent Class Analysis, Cambodia

## Abstract

**Background:**

Cambodia is experiencing a demographic shift likely to increase the burden of noncommunicable diseases (NCDs). Identifying patterns of risk factors among adults can contribute to efforts to effectively target and prevent these chronic diseases. This study aims to examine latent classes of population risk based on behavioral and metabolic risk factors for NCDs in Cambodia.

**Methods:**

Data from 5275 respondents aged 18 and older from the 2023 Cambodian World Health Survey Plus were used for analysis. Latent class analysis identified distinct classes of individuals with similar behavioral and metabolic risk factors. Indicator variables included tobacco and alcohol use, fruit and vegetable diet, physical activity, body mass index, blood pressure, blood glucose, and total cholesterol and triglyceride levels. Multinomial logistic regression was employed to predict latent class membership based on sociodemographic characteristics.

**Results:**

Three distinct latent classes were identified: “alcohol users with lower metabolic risk” (37.8%), “substance users with compounding unhealthy behaviors” (15.0%), and “alcohol users with higher metabolic risk” (47.2%). Men, older adults, and individuals with lower education were more likely to be substance users with compounding unhealthy behaviors and alcohol users with higher metabolic risks.

**Conclusions:**

These findings highlight the need for targeted public health strategies to address the combined impact of multiple risk factors, particularly among men, older adults, and individuals with lower education levels.

**Supplementary Information:**

The online version contains supplementary material available at 10.1007/s44197-025-00464-0.

## Introduction

The global population aged 60 years or older is projected to almost double by 2050, reaching 2.1 billion [1]. Approximately 63% of this increase will come from Asian regions [1], including Southeast Asia, where countries like Cambodia are rapidly transitioning toward an aging society. In 2019, nearly 9% of Cambodia’s population was aged 60 years or older, an estimate projected to exceed 23% by 2050 [2]. This demographic shift is driven by declining fertility rates and increasing life expectancies [2]. An aging population is often accompanied by a rising burden of disease, with a greater prevalence of noncommunicable diseases (NCDs) and associated mortality. As of 2021, 61% of all deaths in Cambodia were attributed to NCDs, with the leading causes being cardiovascular disease, diabetes, chronic respiratory disease, digestive diseases, and cancer [3].

Behavioral risk factors, such as tobacco use, alcohol consumption, poor dietary habits, and physical inactivity, are consistently linked to an increased risk of NCDs. Globally, awareness is increasing about the social and commercial determinants of these risks, and the actions and policies that can shift these drivers of risk [4, 5]. Despite policies and initiatives to mitigate NCD risk factors in Cambodia, they remain a significant public health concern. The 2021 National Adult Tobacco Survey reported that 25% of men and 2% of women aged 15 or older smoke cigarettes, while chewing tobacco is more common among women in rural areas (6.5%) than men (0.3%) [6]. The 2022 Cambodian Demographic Health Survey reported that 64% of men and 16% of women consume alcohol regularly [7]. Diet also contributes to NCD risk, with the World Health Organization (WHO) recommending a daily intake of at least five servings of fruits and vegetables to reduce the risk of NCDs such as cardiovascular disease and certain cancers. However, a recent risk factor survey found that 80% of Cambodian adults fail to meet this dietary recommendation [8]. Most Cambodian adults achieve the recommended levels of physical activity (150–300 min of moderate aerobic activity per week), primarily through work–related activities [8, 9]. Physical inactivity is more common among the older population, with over 15% of adults aged 60–69 failing to meet activity guidelines. Additionally, 85% of women and 33% of men aged 18–69 reported never engaging in vigorous physical activity [8]. Unhealthy lifestyle behaviors often co–occur, amplifying their cumulative impact on health. One study found that 16% of the adult Cambodian population exhibited a combination of smoking, current alcohol consumption, and insufficient physical activity [10]. Evidence indicates that the risk of all–cause mortality is 1.8 times higher among individuals with an accumulation of unhealthy behaviors, such as daily smoking and drinking, low fruit and vegetable intake, and inactivity [11]. Examining multiple concurrent risk factors reflects reality and is essential to understanding their combined impact on health systems and health outcomes, including the progression to NCDs.

In addition to behavioral risk factors, certain metabolic conditions cluster together and significantly increase the risk of developing heart disease, stroke, and type 2 diabetes. These conditions, collectively known as metabolic syndrome, include elevated blood pressure, increased blood glucose, abnormal cholesterol and triglyceride levels, and abdominal obesity [12]. According to a risk factor survey in Cambodian adults (18–69 years), approximately 17% had or were being treated for high blood pressure, and 6.3% had elevated blood glucose levels [8]. The same survey reported that one in four adults had high total cholesterol or were on medication for elevated cholesterol. In Cambodia, hypertension and central obesity are the most prevalent concurrent metabolic risk factors, similar to findings in Myanmar [13]. In contrast, total cholesterol and central obesity were the dominant risk factors in Laos, while hypertension and total cholesterol were the most significant in Viet Nam, highlighting some regional variation within Southeast Asia [13]. However, previous studies have largely focused on individual risk factors, or cumulative number, without considering how multiple concurrent risk factors may be impacting health outcomes. This limits a government’s ability to design effective prevention strategies that target high-risk groups. To address this knowledge gap, Latent Class Analysis (LCA) was employed to identify and explore more nuanced mixtures of behavioral and metabolic risk factors within the adult Cambodian population.

LCA identifies unobserved groups of individuals with similar characteristics based on responses to observed variables. LCA has been used by previous studies to classify individuals into distinct groups with similar risk profiles based on their behavioral and metabolic risk factors [11, 14]. It is also essential to examine how structural factors, such as income, education, occupation, and area of residence, shape individual risk factors and health, as outlined in the WHO’s Conceptual Framework for Action on the Social Determinants of Health (CSDH) [15]. By integrating the CSDH framework, the study identifies socially patterned inequalities in these risk profiles, informing efforts to reduce health disparities. This study aims to (i) identify subgroups of the adult Cambodian population at risk for NCDs based on behavioral and metabolic risk factors using LCA; and (ii) examine whether social determinants such as education, gender, geographical location, marital status, and household wealth predict the identified latent classes.

## Methods

### Data Source and Sampling Process

Data were analyzed from the 2023 Cambodian World Health Survey Plus (WHS +), a population-based, cross-sectional survey conducted to assess the health status, behaviors, and risk factors of adults living in Cambodia. The survey was implemented by the National Institute of Public Health (NIPH), with technical support from the World Health Organization. WHS + Cambodia targeted adults aged 18 years or older residing in individual households who could provide informed consent. A three-stage cluster sampling design was employed. First, 276 villages were selected from a sampling list of 14,568 in all 25 provinces using probability proportional to village size. Second, 44 households were randomly chosen within the village, with 22 anticipated as eligible based on pre-testing, which indicated that approximately 50% of the buildings contained eligible households. Finally, all household members were listed, with one individual aged 18 or older per household randomly selected to complete the individual questionnaire. If the selected individual could not participate, no replacement was made. The recruitment process identified 6,154 eligible households, resulting in 5,275 individual respondents, with an overall response rate of 85.7%.

### Data Collection

A computer-assisted personal interviewing (CAPI) program supported data collection by 14 survey team leaders, 65 trained interviewers, and 14 trained biomarker collectors between 12 March and 31 May 2023. The survey consisted of standardized modules in household and individual questionnaires, including validated interview questions on sociodemographic characteristics, health status, and health behaviors. Additionally, anthropometric measurements (height, weight, waist, and hip circumferences), performance tests, and blood sample testing were conducted.

### Study Variables

#### Behavioral Factors

The survey included questions about three forms of tobacco use: smoking tobacco, smokeless tobacco, and e–cigarettes. Respondents were shown images of products in each category and asked if they currently used any of them. Those who responded “not at all” were categorized as non–current tobacco users, while those who answered “daily or less than daily” for any of the three tobacco forms were classified as current tobacco users. Those who answered “don’t know” were coded as missing. Alcohol consumption was assessed by asking respondents if they had consumed alcohol in the past 30 days. Those who had were categorized as current alcohol consumers, while those who had not were non–current alcohol consumers [7]. Fruit and vegetable intake was categorized based on WHO recommendations for daily servings [16]. Respondents who reported consuming at least five servings of fruits and vegetables per day were classified as having adequate intake, while those consuming fewer than five servings were classified as having inadequate intake. Physical activity was measured within WHS + through incorporating the WHO Global Physical Activity Questionnaire (GPAQ), which assessed self–reported physical activity across three domains: occupational, transport, and recreational [17]. Participants reported the duration, frequency, and intensity of activities, categorized into six types: vigorous work, moderate work, daily transport/travel, vigorous recreation, moderate recreation, and sitting. The metabolic equivalent of task (MET) was used to quantify activity intensity. The MET values were used to quantify activity intensity: 8 METs for vigorous activities (work and recreation), 4 METs for moderate activities (work, recreation, and travel), and 0 METs for sitting. Total physical activity was calculated as the sum of MET minutes per week across all domains. Participants with ≥ 600 MET minutes/week were classified as physically active, while those with < 600 MET minutes/week were classified as physically inactive [17].

#### Metabolic Factors

Using Asian–Pacific criteria, a body mass index (BMI) of 23–25 kg/m^2^ was classified as overweight and > 25 kg/m^2^ as obese [18]. For analysis, BMI > 23 kg/m^2^ was categorized as overweight/obesit*y.* Blood pressure was measured three times while the participants were seated and relaxed, with the mean of the last two readings used for analysis. An elevated blood pressure variable was created based on the systolic/diastolic value threshold of ≥ 130/≥ 85 mmHg (the National Cholesterol Education Program/NCEP III guidelines) [12]. Finger–prick non-fasting blood samples were used to measure glycated hemoglobin (HbA1c), cholesterol and triglyceride levels. HbA1c levels ≥ 6.5% were categorized as elevated, while 4.0–6.4% was normal [19]. Total cholesterol levels ≥ 240 mg/dL and triglyceride ≥ 150 mg/dL were considered as elevated [12].

#### Sociodemographic Variables

Survey respondents reported information on age, gender, marital status, education level, and residence area. Gender was categorized as man, woman, and non-binary in the survey, and age was grouped into five groups (18–39, 40–49, 50–59, 60–69, and ≥ 70 years). Indicators for marital status used three categories (currently married, never married, and divorced or widowed), while education level was grouped into five: at least high school, completed secondary, completed primary, incomplete primary school, and never schooling. Household economic status was determined using the Demographic and Health Surveys (DHS) Wealth Index guidelines [20], including home ownership, construction materials, cooking fuel, sanitation, water sources, handwashing facilities, and asset ownership. The Kaiser–Meyer–Olkin (KMO) test assessed suitability for Principal Component Analysis (PCA), with values of 0.7–0.9 indicating good to excellent suitability. The first principal component, explaining the most variance, was used to construct the wealth index, ranked into quintiles (Q1 poorest to Q5 wealthiest). Urban and rural indices were calculated separately and combined into a single variable. Detailed variables’ definition and measurements are available in Additional file 1.

#### Statistical Analysis

The data analysis in this study was conducted in two main steps: (i) identifying latent classes based on behavioral and metabolic risk patterns, (ii) examining associations between sociodemographic variables and latent class membership. All analyses were performed using the *gsem* package in Stata/Standard Edition (SE) 18.0 (StataCorp, College Station, TX, USA).

In the first step, LCA was used to categorize individuals in the sample (n = 5275) into mutually exclusive groups (latent classes) based on their responses to behavioral and metabolic risk indicators. Prior to analysis all indicators were assessed for collinearity. Models with one to six classes (k-1 to k-6) were explored and evaluated based on a balance of statistical fit and interpretability, ensuring that each latent class was conceptually meaningful. Specifically, models were compared using the Akaike Information Criterion (AIC), Bayesian Information Criterion (BIC), consistent Akaike Information Criterion (CAIC), and entropy. Lower values of AIC, BIC, and CAIC, along with higher entropy values, were indicative of a better fit [21]. Models were also evaluated based on the size of the smallest classes and class separation [22]. Once the best-fitting model was determined, latent class membership and conditional item-response probabilities were identified. Classes were named based on patterns of item-response probabilities within each class. Fixed thresholds in LCA do not apply for interpreting item-response probabilities. Instead, class labels were assigned based on how each indicator compared to both the other classes and the overall sample. Then, each individual was assigned to a latent class based on their maximum posterior probability [21]. Missing data were handled using full information maximum likelihood (FIML) estimation.

In the second step, the association between sociodemographic variables (gender, age, region, marital status, education, and wealth) and latent class membership was analyzed using a multinomial logistic regression model with Class 1 as the reference group. Odds ratios (ORs) with 95% confidence intervals (CIs) were reported, with statistical significance defined as *p* < 0.05. Missing data in the regression analysis (see Additional file 2) were addressed using Multiple Imputation by Chained Equations (MICE) with 20 repetitions, accounting for 24.1% of the sample (*n* = 1270). To allow for comparison between Class 2 and 3, a multinomial regression was also conducted using Class 2 as the reference group (see Additional file 7).

To ensure robustness, probability weights were incorporated in the LCA and multinomial regression. Additionally, a sensitivity analysis was conducted with complete cases only for the LCA (see Additional files 5 and 6).

## Results

Among the 5,275 respondents, most were women (52.2%), aged 18–39 years old (54.2%), residing in rural areas (58.2%), and married (79.7%). No respondent identified as non-binary. Educational attainment was low, with only 14.5% completing at least high school, while 25.9% had incomplete primary education. Most respondents belonged to the lowest wealth quintile (26.2%). Concerning lifestyle behaviors, about 17.6% reported currently using tobacco, and nearly 39.7% had consumed alcohol in the past 30 days. Over half of the respondents reported inadequate fruit and vegetable consumption. Around 36% of the sample were classified as physically inactive. 48.2% of the respondents were classified as overweight or obesity. Nearly 31% had elevated blood pressure, while 9.9% of respondents had HbA1c levels ≥ 6.5, 11% had elevated total cholesterol levels, and about 41% had raised triglycerides (Table 1).

**Table 1:** Descriptive statistics of the study sample

Variables	*n* (weighted %)
Total	5275 (100)
Gender	
Women	3658 (52.2)
Men	1617 (47.8)
Age (mean, SD)	47.3 (0.37)
Age group	
18–39	1784 (54.2)
40–49	1087 (17.4)
50–59	1093 (13.2)
60–69	912 (9.1)
70 +	399 (6.1)
Region type	
Urban	2059 (41.8)
Rural	3216 (58.2)
Marital status	
Currently married	4088 (79.7)
Never married	297 (11.6)
Divorced/widowed	890 (8.7)
Education level	
At least high school	446 (14.5)
Completed secondary school	641 (18.2)
Completed primary school	1213 (26.9)
Incomplete primary	1831 (25.9)
Never schooling	1114 (14.5)
Household economic group	
Q5 (wealthiest)	1002 (14.3)
Q4	1011 (16.6)
Q3	1021 (19.6)
Q2	971 (21.6)
Q1 (poorest)	1077 (26.2)
Missing	193 (1.7)
Tobacco use	
Not currently using	4093 (80.3)
Currently using	1055 (17.6)
Missing	127 (2.1)
Alcohol consumption	
Not currently drinking	3574 (48.3)
Currently drinking	1680 (39.7)
Missing	21 (12.1)
Fruit and vegetable intake	
Adequate	2229 (43.0)
Inadequate (< 5 servings daily)	2960 (55.3)
Missing	86 (1.7)
Physical activity	
Active (high and moderate)	3540 (63.9)
Inactive (low)	1735 (36.1)
Body Mass Index	
No overweight or obesity	2175 (44.3)
Overweight/obesity (> 25 kg/m^2)^	2783 (48.2)
Missing	8 (7.5)
Blood pressure	
Normal	3157 (62.5)
Elevated (≥ 130/≥ 85 mmHg)	1835 (30.5)
Missing	283 (7.0)
Hemoglobin A1C (HbA1c)	
Normal	3684 (71.1)
Elevated (≥ 6.5%)	678 (9.9)
Missing	913 (19.0)
Total cholesterol	
Normal	4218 (81.2)
Elevated (≥ 240 mg/dL)	739 (11.0)
Missing	318 (7.8)
Total triglycerides	
Normal	2581 (51.4)
Elevated (≥ 150 mg/dL)	2374 (40.8)
Missing	320 (7.8)

### Latent Class of Behavioral and Metabolic Risk Factors

While the BIC and CAIC reached their lowest values in the 6-class model, further examination revealed that several classes had class membership prevalence below 10%, suggesting overfitting and poor model fit [22]. Additionally, the 6-class model showed poor separation in item-response probabilities, potentially indicating that outliers in a single indicator were disproportionately influencing class formation (see Additional file 8). Given the minimal improvements in AIC, BIC, and CAIC beyond the 3-class model and the added clinical interpretability it offered, the 3-class model was selected as the best fitting model. Notably, the entropy value of 0.850 further supports this choice, indicating strong class separation (Table 2).

**Table 2:** Goodness of fit for latent class models (*n* = 5275)

Model	LL	df	AIC	BIC	CAIC	Entropy
1	−55835263.85	9	111670545.7	111670604.8	111670613.8	NA
2	−54402727.34	19	108805492.7	108805617.5	108805636.5	0.880
**3**	**−54133442.79**	**29**	**108266943.6**	**108267134.1**	**108267163.1**	**0**.**850**
4	−54063548.67	38	108127173.3	108127423.0	108082233.5	0.865
5	−53974184.71	44	107948457.4	107948746.5	108010154.0	0.852
6	−53937727.71	56	107875567.4	107875935.4	107874874.5	0.814

*LL* Log likelihood, *df* Degrees of Freedom, *AIC* Akaike Information Criterion, *BIC* Bayesian Information Criterion, *CAIC* Consistent Akaike Information Criterion

**Fig. 1:**
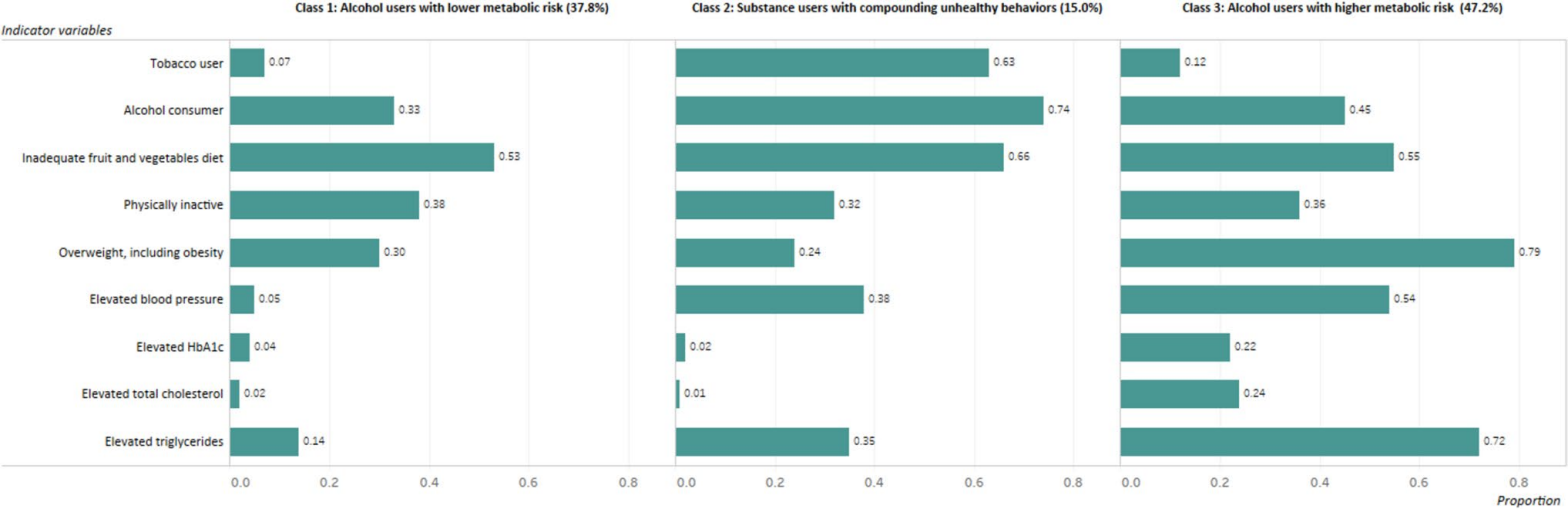
Prevalence of latent classes (%) with conditional item probabilities for each class

As seen in Fig. 1, three distinct classes were identified. Class 1, “alcohol users with lower metabolic risks”, accounted for 37.8% of the sample. The LCA shows, given membership in Class 1, individuals had a lower probability of current tobacco use and a lower probability of elevated metabolic risk factors. Adults in Class 2, “substance users with compounding unhealthy behaviors,” represented 15.0% of the sample. Members of this class were more likely to use tobacco and alcohol, eat inadequate fruit and vegetables, and be physically inactive. Class 3, “alcohol users with higher metabolic risk,” consisted of 47.2% of the sample and was characterized by elevated metabolic risk factors, including high blood pressure, HbA1c, total cholesterol and triglycerides.

### Association Between Sociodemographic Characteristics and Latent Class Membership

Table 3 shows the weighted relative risk ratios (RRR) and 95% confidence interval (CI) for Class 2 “substance users with compounding unhealthy behaviors” and Class 3 “alcohol users with higher metabolic risk”, compared to Class 1 “alcohol users with lower metabolic risk”. Class 1, characterized by the lowest prevalence of risk factors compared to other classes, served as the reference group. Full and crude results of multinomial regression in Additional files 3 and 4.

**Table 3:** Weighted multinomial logistic regression of sociodemographic characteristics predicting latent class membership in relation to Class 1

Sociodemographic variables	Class 2			Class 3		
	*Substance users with compounding unhealthy behaviors*			*Alcohol users with higher metabolic risk*		
	RRR	(95% CI)	*p*–value	RRR	(95% CI)	*p*–value
Gender (ref women)						
Men	14.33	(9.86–20.8)	< 0.001	1.87	(1.50–2.33)	< 0.001
Age group (ref 18–39 years)						
40–49	1.25	(0.76–2.06)	0.379	1.90	(1.42–2.54)	< 0.001
50–59	2.67	(1.65–4.32)	< 0.001	3.13	(2.28–4.40)	< 0.001
60–69	1.43	(0.80–2.59)	0.228	2.51	(1.71–3.68)	< 0.001
70 +	1.78	(0.86–3.71)	0.122	2.11	(1.31–3.39)	0.002
Residence area (ref urban)						
Rural	1.07	(0.74–1.55)	0.717	0.62	(0.50–0.77)	< 0.001
Marital status (ref currently married)						
Never married	0.48	(0.25–0.86)	0.037	0.51	(0.35–0.78)	0.001
Divorced/Widowed	1.14	(0.65–2.04)	0.636	1.10	(0.79–1.53)	0.583
Education level (ref at least high school)						
Completed secondary	1.50	(0.69–3.28)	0.308	0.94	(0.63–1.39)	0.743
Completed primary	2.26	(1.08–4.74)	0.031	1.48	(1.02–2.15)	0.038
Incomplete primary	2.76	(1.31–5.76)	0.007	1.36	(0.92–1.99)	0.115
Never schooling	2.81	(1.31–6.07)	0.008	1.04	(0.67–1.60)	0.859
Household economic group (ref Q5 wealthiest)						
Q4	1.08	(0.60–1.95)	0.789	0.74	(0.53–1.01)	0.060
Q3	1.43	(0.80–2.55)	0.222	0.71	(0.51–0.97)	0.034
Q2	1.31	(0.72–2.37)	0.375	0.53	(0.38–0.74)	< 0.001
Q1 poorest	2.15	(1.21–3.81)	0.008	0.62	(0.44–0.88)	0.007

*CI* confidence interval, *Ref* reference group, *RRR* relative risk ratio

Reference category for risk factor classes was Class 1 ‘Alcohol users with lower metabolic risk’

Substance users were defined as higher item response probability for tobacco and alcohol use

Compared to alcohol users with lower metabolic risk, men were significantly more likely to be “substance users with compounding unhealthy behavior (RRR 14.33; 95% CI 9.86–20.8) than women. Those aged 50–59 also had a higher risk of belonging to the substance users with compounding unhealthy behaviors class (RRR 2.67; 95% CI 1.65–4.32). Never marrying was associated with a lower risk compared to being married (RRR 0.48; 95% CI 0.25–0.86). Educational attainment was inversely related, with those with no formal schooling having the highest risk (RRR 2.81; 95% CI 1.31–6.07). Similarly, individuals from the poorest households had an increased risk of being substance users with compounding unhealthy behaviors (RRR 2.15; 95% CI 1.21–3.81) relative to being alcohol users with lower metabolic risk.

Comparing alcohol users with higher metabolic risk to alcohol users with lower metabolic risk, men were more likely to belong to the class with higher metabolic risk compared to women (RRR 1.87; 95% CI 1.50–2.33). The likelihood of being alcohol users with higher metabolic risk increased with age, with those aged 50–59 showing the highest risk (RRR 3.13; 95% CI 2.28–4.40). In contrast, individuals living in rural areas (RRR 0.62; 95% CI 0.50–0.77) and those who had never married (RRR 0.51; 95% CI 0.35–0.78) were less likely to be alcohol users with higher metabolic risk. Educational attainment was a significant factor associated with being alcohol users with higher metabolic risk for those who had only completed primary school (RRR 1.48; 95% CI 1.02–2.15). However, respondents from the poorest households were less likely to be alcohol users with higher metabolic risk (RRR 0.62; 95% CI 0.44–0.88) compared to alcohol users with low metabolic risk. When using Class 2 “substance users with compounding unhealthy behaviors” as a reference group, the results shows that men, individuals living in rural areas, and those with no schooling and of lower wealth were less likely to belong to the alcohol users with higher metabolic risk class. However, individuals aged 60–69 years old were more likely to be alcohol users with higher metabolic risk (Class 3) than substance users with compounding unhealthy behaviors (Class 2) (see Additional file 7).

A sensitivity analysis was conducted using a complete case analysis (Additional files 5 and 6), showing a comparable three-class solution, with similar class profiles and sample distribution. The entropy value of 0.859 also indicates strong class separation. This supports the stability of the identified latent classes despite missing data.

## Discussion

This study identified three distinct latent classes representing patterns of behavioral and metabolic risk factors for NCDs in a population-based sample of Cambodian adults. Specifically, Class 1 was characterized by alcohol users with lower metabolic risks, Class 2 included substance users who engaged in multiple unhealthy behaviors, and Class 3 comprised alcohol users with higher metabolic risks. The high homogeneity within classes and good separation between classes emphasize the distinct clustering of behavioral and metabolic NCD risk factors in this population. Men, 50–59-year-olds, and those with lower education levels were more likely to be substance users with increased unhealthy behaviors (Class 2) and alcohol users with higher metabolic risk (Class 3). Respondents living in rural areas had a lower probability of being alcohol users with higher metabolic risks. Those who had never married were less likely to belong to belong to either the substance users with compounding unhealthy behaviors or the alcohol users with higher metabolic risk classes. Individuals from lower socioeconomic groups were more likely to be substance users with increased unhealthy behaviors but less likely to be alcohol users with higher metabolic risks compared to alcohol users with lower metabolic risks.

Substance users with compounding unhealthy behaviors demonstrated the highest likelihood of combined behavioral risk factors, including tobacco and alcohol use, insufficient fruit and vegetable consumption, and physical inactivity. A previous study reported similar findings when assessing the prevalence of multiple risk factors in Cambodia [10]. Our study advances this understanding by uncovering distinct subgroups based on unobserved and underlying patterns of co-occurring behaviors using LCA. The demographic profile of substance users with compounding unhealthy behaviors was characterized by a higher likelihood of being a man, aged 50–59, uneducated, and from a poorer socioeconomic group. Patterns of risky behaviors in Cambodia and other Southeast Asian countries tend to be higher among men and increase with age [10, 13], aligning with the characteristics identified in this study, although the likelihood of being in the class with more unhealthy behaviors was not significant for ages 60 years and older.

More than half of the sample were alcohol users with higher metabolic risks, showing significantly high probabilities of elevated metabolic markers, including hypertension, dyslipidemia, elevated HbA1c, and obesity, which are the hallmarks of metabolic syndrome [12]. Individuals living in rural areas had a lower likelihood of belonging to this class in this analysis. Urbanization has been linked to the rising prevalence of NCDs in Southeast Asian countries [23], and in urban Cambodia, approximately 60% of men and 52% of women reportedly have metabolic syndrome [24]. Additionally, drinking alcohol has been associated with a higher likelihood of having metabolic syndrome among both men and women living in urban Cambodia [25]. However, the similarity in alcohol, dietary, and physical activity patterns between the two classes underscores that metabolic risk cannot be entirely explained by commonly targeted lifestyle factors, suggesting the importance of potentially unmeasured social or genetic determinants in shaping metabolic health disparities. 

Structural determinants, such as disparities in wealth and education, were associated with class membership. Lower-educated and poorer socioeconomic groups faced higher risks of being substance users with compounding unhealthy behaviors. A systematic review in low- and lower-middle-income countries observed similar trends, where individuals from lower socioeconomic backgrounds were more likely to use alcohol and tobacco, and have lower intake of fruits and vegetables compared to those from higher socioeconomic groups [26]. In Cambodia, higher levels of education and wealth are associated with a lower likelihood of using tobacco [27]. While education was not significantly associated with membership in the class with higher metabolic risks for the lowest education categories, individuals with primary education only were more likely to belong to this group compared to those who had completed high school. Individuals with limited education may face the challenge of managing metabolic risk factors due to delayed NCD diagnosis, higher treatment attrition, and poor compliance with prescribed interventions [28]. Those with insufficient health literacy may also be more susceptible to the commercial determinants of health, impeding an individual's ability to manage their health effectively [29]. Cambodian adults with only elementary school education have previously demonstrated lower self-efficacy regarding NCD prevention, often expressing lower levels of agreement about health behaviors such as attending health screening or seeking necessary information to prevent these diseases [30]. Our findings highlight that while structural factors such as wealth and education remain strong predictors of behavioral risk clustering, the relationship between education and metabolic risk may be more complex, potentially moderated by factors such as access to healthcare, health literacy, or urban exposure.

### Public Health Implications

The results from this study have the potential to inform NCD prevention and screening efforts, especially since early detection of NCDs in Cambodia is still inadequate [31]. The coexistence of unhealthy behaviors and metabolic risk factors highlights the need for public health strategies that screen for and address multiple, interacting risk factors rather than focusing on isolated ones. Identifying latent classes enables more precise targeting of interventions by uncovering distinct combinations of behavioral and metabolic risks that may remain hidden when examining individual risk factors in isolation. This approach supports the development of tailored prevention strategies that address the specific needs and contexts of high-risk subgroups, thereby enhancing intervention effectiveness. A recent meta-analysis found a positive linear effect between adherence to multiple behavioral recommendations and improved health outcomes, such as cardiovascular health and smoking cessation [32]. Furthermore, low perceived self-efficacy has been identified as a significant barrier to effective NCD prevention in Cambodia [30], emphasizing the need to strengthen individuals' confidence and knowledge about health maintenance. Research indicates that individuals diagnosed with a disease by a doctor often report fewer barriers to NCD prevention following medical examinations [30]. This presents an opportunity to utilize medical interactions as a platform to enhance self–efficacy.

The observed disparities in class membership by socioeconomic status and education highlight the critical role of structural inequalities in shaping NCD risk in Cambodia. Individuals from lower-income households and with lower education attainment were disproportionately represented in classes characterized by multiple unhealthy behaviors, likely reflecting broader inequities in access to health-promoting resources, accurate health information, and affordable preventive services. Tackling such disparities requires a dual approach: targeted health education initiatives to raise NCD awareness and empower individuals, coupled with comprehensive structural interventions. These should intensify policies that expand access to quality education, promote heathier work and living environments, strengthen social protection systems, and remove financial and logistic barriers to preventive care. Without addressing these upstream determinants, efforts to reduce NCD risk are unlikely to achieve equitable and sustainable impact.

### Strengths and Limitations

Our study utilized LCA on a recently collected population–based sample from WHS +, making it the first application of this approach to NCD risk factors in the Cambodian context. LCA has been widely used in other studies to identify subgroups of people with NCD risk factors [11, 14], and its person–centered approach is crucial for tailoring targeted interventions and personalized approaches in public health. WHS + integrates validated behavioral, metabolic, and clinical health measures, making it a powerful tool for evidence-based research. Given that detailed general population health surveys are less common in Cambodia, the high response rate (87.5%) underscores WHS + effectiveness in gathering reliable, nationally representative health data. Moreover, our use of sampling probability weights in all analyses enhances the generalizability of findings to the national adult population by accounting for the complex survey design, further mitigating the risk of bias.

Several limitations should be mentioned. The cross–sectional design limits the ability to examine causal or temporal relationships between determinants of classes. Longitudinal data could enable latent transitional analysis to investigate changes in behavior patterns over time. Furthermore, assigning individuals to latent classes based on posterior probabilities introduces classification errors, although our entropy value (0.858) indicates a good separation of individuals into classes. The reliance on self-reported data for lifestyle behaviors introduces the potential for recall bias, which may affect the accuracy of reported health behaviors. Moreover, findings may not be fully generalizable to the broader population, as class categorization depends on the indicator variables used in the analysis. Although the survey had a high response rate, non-response may still introduce bias if non-participants differ systematically in health status or socioeconomic background or were known as missing not at random. The pattern of non-respondents was not known. If missing not at random occurred, this could lead to over- or underestimation of specific risk profiles. However, the combination of a rigorous multistage sampling design, high response rate, and the application of probability weights likely reduced the extent of this bias.

## Conclusion

The study identified three latent classes of behavioral and metabolic risk factors for NCDs in an adult Cambodian population, each with distinct sociodemographic profiles. The majority of the participants belonged to the class characterized by elevated metabolic levels. These results can guide targeted interventions and personalized management strategies. Gender, age, wealth, and educational attainment were strong predictors of class membership, highlighting the role of social determinants in shaping health risks. Future research should delve into the complexities of at-risk groups beyond these factors, examining how social position, stress, and other contextual influences contribute to the NCD burden in Cambodia. Investigating health–related decision–making processes and access to NCD prevention and care services will provide critical insights into the disparities underlying these risk factors and help develop more equitable public health strategies.

## Supplementary Information

Below is the link to the electronic supplementary material.

Supplementary file1 (DOCX 19 KB)

Supplementary file2 (DOCX 14 KB)

Supplementary file3 (DOCX 22 KB)

Supplementary file4 (DOCX 20 KB)

Supplementary file5 (DOCX 14 KB)

Supplementary file6 (DOCX 15 KB)

Supplementary file7 (DOCX 20 KB)

Supplementary file8 (DOCX 15 KB)

## Declarations

### Data Availability

The public version of the WHS + data will be available upon reasonable request through https://apps.who.int/healthinfo/systems/surveydata/index.php/collections/central.

## References

[1] United Nations, Department of Economic and Social Affairs. *World **Social **Report 2023: **Leaving No One Behind in an Ageing World.* [Internet]. New York: United Nations. 2023. Report No.: ST/ESA/379. Sales No.: E.23.IV.2. Available from: https://www.un.org/development/desa/dspd/world-social-report/2023.html

[2] National Institute of Statistics (Cambodia). Final General population census of Cambodia 2019. [Internet]. Phnom Penh. National Institute of Statistics. 2020. Available from: https://www.nis.gov.kh/nis/Census2019/Final%20General%20Population%20Census%202019-English.pdf

[3] Institute for Health Metrics and Evaluation. Global burden of disease study 2021. Available from: https://vizhub.healthdata.org/gbd-results/. Accessed 08 Feb 2025.

[4] Marmot M, Bell R. Social determinants and non-communicable diseases: time for integrated action. BMJ. 2019;364:l251. 30692093 10.1136/bmj.l251 PMC6348404

[5] Lacy-Nichols J, Nandi S, Mialon M, McCambridge J, Lee K, Jones A, et al. Conceptualising commercial entities in public health: beyond unhealthy commodities and transnational corporations. Lancet. 2023;401(10383):1214–28. 36966783 10.1016/S0140-6736(23)00012-0

[6] Launching of National Adult Tobacco Survey of Cambodia (NATSC) 2021 Country Report [Internet]. HACC. Health action coordinating committee; 2023. Available from: https://hacccambodia.org/launching-of-national-adult-tobacco-survey-of-cambodia-natsc-2021-country-report/. Accessed 10 December 2024.

[7] Um S, Heng S, Mok S, Chamroen P, Sopheab H. Determinants of alcohol consumption among men and women aged 15–49 years in Cambodia: evidence from the Cambodia demographic and health survey 2021–2022. Drug Alcohol Rev. 2025;44(2):448–58. 39686585 10.1111/dar.13994

[8] Ministry of Health (Cambodia). *Prevalence of non–communicable disease risk factors in Cambodia STEPS survey country report. 2023*. [Internet]. Phnom Penh: Ministry of Health. 2023. Available from: https://cdn.who.int/media/docs/default-source/ncds/ncd-surveillance/data-reporting/cambodia/cambodia-2023-steps-report-english-final.pdf

[9] WHO guidelines on physical activity and sedentary behaviour: at a glance. [Internet]. Geneva: World Health Organization. Licence: CC BY–NC–SA 3.0 IGO. 2020. Available from: https://iris.who.int/server/api/core/bitstreams/faa83413-d89e-4be9-bb01-b24671aef7ca/content 33369898

[10] Ma M, He L, Wang H, Tang M, Zhu D, Sikanha L, et al. Prevalence and clustering of cardiovascular disease risk factors among adults along the Lancang-Mekong River: a cross-sectional study from low– and middle-income countries. Glob Heart. 2024;19(1):35. 38638126 10.5334/gh.1319 PMC11025572

[11] Kukreti S, Yu T, Chiu PW, Strong C. Clustering of modifiable behavioral risk factors and their association with all-cause mortality in Taiwan’s adult population: a latent class analysis. Int J Behav Med. 2022;29(5):565–74. 34775543 10.1007/s12529-021-10041-x PMC9525409

[12] National Cholesterol Education Program (NCEP) Expert Panel on Detection, Evaluation, and Treatment of High Blood Cholesterol in Adults (Adult Treatment Panel III). Third report of the national cholesterol education program (NCEP) expert panel on detection, evaluation, and treatment of high blood cholesterol in adults (Adult Treatment Panel III) final report. Circulation. 2002;106(25):3143–421. 12485966

[13] Biswas T, Townsend N, Gupta RD, Ghosh A, Rawal LB, Mørkrid K, et al. Clustering of metabolic and behavioural risk factors for cardiovascular diseases among the adult population in South and Southeast Asia: findings from WHO STEPS data. Lancet Reg Health. 2023;12:100164. 10.1016/j.lansea.2023.100164 PMC10305930 37384055

[14] Mkuu RS, Gilreath TD, Barry AE, Nafukho FM, Rahman J, Chowdhury MAB, et al. Identifying individuals with multiple non–communicable disease risk factors in Kenya: a latent class analysis. Public Health. 2021;198:180–6. 34461453 10.1016/j.puhe.2021.07.031

[15] World Health Organization. *A conceptual framework for action on the social determinants of health.* [Internet]. Discussion paper no. 2. Geneva: World Health Organization. 2010; 76. Available from: https://iris.who.int/handle/10665/44489

[16] World Health Organization. *Diet, nutrition and the prevention of chronic diseases: report of a joint WHO/FAO expert consultation*. [Internet]. WHO technical report series, No. 916. Geneva: Switzerland. 2003. Available from: https://iris.who.int/server/api/core/bitstreams/c4f12939-5f55-4b75-b6f9-283024956b3f/content

[17] Armstrong T, Bull F. Development of the world health organization global physical activity questionnaire (GPAQ). J Public Health. 2006;14(2):66–70.

[18] Tham KW, Abdul Ghani R, Cua SC, Deerochanawong C, Fojas M, Hocking S, et al. Obesity in South and Southeast Asia–a new consensus on care and management. Obes Rev. 2023;24(2):e13520. 36453081 10.1111/obr.13520 PMC10078503

[19] World Health Organization. *Use of glycated haemoglobin (HbA1c) in the diagnosis of diabetes mellitus: abbreviated report of a WHO consultation*. [Internet]. Geneva.: World Health Organization. 2011. Available from: https://iris.who.int/server/api/core/bitstreams/db9b9d3d-f95e-4797-9d2b-c78dcef0133f/content 26158184

[20] Rutstein SO. Steps to construct new DHS wealth index. 2015. Available from: https://dhsprogram.com/programming/wealth%20index/Steps_to_constructing_the_new_DHS_Wealth_Index.pdf. Accessed 10 November 2024.

[21] Weller BE, Bowen NK, Faubert SJ. Latent class analysis: a guide to best practice. J Black Psychol. 2020;46(4):287–311.

[22] Sinha P, Calfee CS, Delucchi KL. Practitioner’s guide to latent class analysis: methodological considerations and common pitfalls. Crit Care Med. 2021;49(1):e63-79. 33165028 10.1097/CCM.0000000000004710 PMC7746621

[23] Angkurawaranon C, Jiraporncharoen W, Chenthanakij B, Doyle P, Nitsch D. Urbanization and non–communicable disease in Southeast Asia: a review of current evidence. Public Health. 2014;128(10):886–95. 25369353 10.1016/j.puhe.2014.08.003

[24] Tamaoki M, Honda I, Nakanishi K, Cheam S, Okawada M, Sakakibara H. Prevalence of metabolic syndrome and its components in urban Cambodia: a cross-sectional study. J Epidemiol Glob Health. 2022;12(3):224–31. 35947272 10.1007/s44197-022-00053-5 PMC9470791

[25] Tamaoki M, Honda I, Nakanishi K, Nakajima M, Cheam S, Okawada M, et al. Lifestyle factors associated with metabolic syndrome in urban Cambodia. Int J Environ Res Public Health. 2022;19(17):10481. 36078197 10.3390/ijerph191710481 PMC9518541

[26] Allen L, Williams J, Townsend N, Mikkelsen B, Roberts N, Foster C, et al. Socioeconomic status and non–communicable disease behavioural risk factors in low–income and lower–middle–income countries: a systematic review. Lancet Glob Health. 2017;5(3):e277-89. 28193397 10.1016/S2214-109X(17)30058-X PMC5673683

[27] Sreeramareddy CT, Pradhan PMS, Mir IA, Sin S. Smoking and smokeless tobacco use in nine South and Southeast Asian countries: prevalence estimates and social determinants from demographic and health surveys. Popul Health Metrics. 2014;12:22. 10.1186/s12963-014-0022-0 PMC4151025 25183954

[28] Jacobs B, Men C, Bigdeli M, Hill PS. Limited understanding, limited services, limited resources: patients’ experiences with managing hypertension and diabetes in Cambodia. BMJ Glob Health. 2017;2:e000235. 29291130 10.1136/bmjgh-2016-000235 PMC5717921

[29] Coughlin SS, Vernon M, Hatzigeorgiou C, George V. Health literacy, social determinants of health, and disease prevention and control. J Environ Health Sci. 2020;6(1):3061. 33604453 PMC7889072

[30] Bae SH, Hwang O, Jeong J, Yang Y. Risk perceptions of noncommunicable diseases among Cambodian adults: a cross-sectional study. J Korean Acad Community Health Nurs. 2022;33(2):259.

[31] Te V, Chhim S, Buffel V, Van Damme W, van Olmen J, Ir P, et al. Evaluation of diabetes care performance in Cambodia through the cascade-of-care framework: cross-sectional study. JMIR Public Health Surveill. 2023;9:e41902. 37347529 10.2196/41902 PMC10337437

[32] Zhang AL, Liu S, White BXL, Iu XC, Durantini M, Chan MS, et al. Health–promotion interventions targeting multiple behaviors: a meta–analytic review of general and behavior–specific processes of change. Psychol Bull. 2024;150(7):798–838. 38913732 10.1037/bul0000427 PMC11960000

