# Supplementary material for: Identifying Subgroups At-Risk for Noncommunicable Diseases in Cambodia: A Latent Class Analysis of Behavioral and Metabolic Risk Factor Patterns
Source: J Epidemiol Glob Health. 2025 Oct 13;15(1):119. doi: 10.1007/s44197-025-00464-0 (PMC12518195; doi:10.1007/s44197-025-00464-0)
Supplement: Supplementary file 1 — Supplementary file1 (DOCX 19 KB) [file 44197_2025_464_MOESM1_ESM.docx]

**Additional Table A1**. Variables’ definition and measurement

| **Variables** | **Original variables: description** | **Variables’ measurement** |
| --- | --- | --- |
| **Behavioural risk factors** | | |
| Tobacco use | **Q3001**. Do you currently smoke any of the tobacco products that I mentioned? | 0: Non–current user (not at all)  1: Current tobacco user (daily/less then daily) |
| Alcohol consumption | **Q3100**. Have you ever consumed a drink that contains alcohol (such as beer, wine, spirits)?  [If Q3100 = Yes]  **Q3103**. Have you consumed alcohol in the last 30 days? | 0: Non–alcohol user (abstainer/did not consume in the last 30 days) 1: Current alcohol user (consumed in the last 30 days) |
| Fruit and vegetable intake | **Q3300**. How many servings of fruit do you  eat on a typical day?  **Q3301**. How many servings of vegetables  do you eat on a typical day? | The number of servings of fruit and vegetables were summed to obtain the total number of servings.  0: Adequate (≥ 5 servings daily) 1: Inadequate (< 5 servings daily) |
| Physical activity | **Q3401**. Does your work involve vigorous–intensity activity that causes large increases in breathing or heart rate, [like heavy lifting, digging or chopping wood] for at least 10 minutes continuously?  **Q3402**. In a typical week, on how many days do you do vigorous–intensity activities as part of your work?  **Q3403hh**. How much time do you spend doing vigorous–intensity activities at work on a typical day? (hours).  **Q3403mm**. How much time do you spend doing vigorous–intensity activities at work on a typical day? (minutes).  **Q3404**. Does your work involve moderate–intensity activity that causes small increases in breathing or heart rate [such as brisk walking, carrying light loads, cleaning, cooking, or washing clothes] for at least 10 minutes continuously?  **Q3405**. In a typical week, on how many days do you do moderate–intensity activities as part of your work?  **Q3406hh**. How much time do you spend doing moderate–intensity activities at work on a typical day? (hours)  **Q3406mm**. How much time do you spend  doing moderate–intensity activities at work  on a typical day? (minutes)  *Travel to and from places*  **Q3407**. Do you walk or use a bicycle (pedal cycle) for at least 10 minutes continuously to get to and from places?  **Q3408**. In a typical week, on how many days do you walk or bicycle for at least 10 minutes continuously to get to and from places?  **Q3409hh**. How much time would you spend walking or bicycling for travel on a typical day? (hours)  **Q3409mm**. How much time would you spend walking or bicycling for travel on a typical day? (minutes)  *Recreational activities*  **Q3410**. Do you do any vigorous intensity sports, fitness or recreational (leisure) activities that cause large increases in breathing or heart rate [like running or football], for at least 10 minutes continuously  **Q3411**. In a typical week, on how many days do you do vigorous intensity sports, fitness or recreational (leisure) activities?  **Q3412hh**. How much time do you spend doing vigorous intensity sports, fitness or recreational activities on a typical day? (hours)  **Q3412mm**. How much time do you spend doing vigorous intensity sports, fitness or recreational activities on a typical day? (minutes)  **Q3413**. Do you do any moderate–intensity sports, fitness or recreational (leisure) activities that causes a small increase in breathing or heart rate [such as brisk walking, cycling or swimming] for at least 10 minutes at a time?  **Q3414**. In a typical week, on how many days do you do moderate–intensity sports, fitness or recreational (leisure) activities?  **Q3415hh**. How much time do you spend doing moderate intensity sports, fitness or recreational (leisure) activities on a typical day? (hours)  **Q3415mm**. How much time do you spend doing moderate intensity sports, fitness or recreational (leisure) activities on a typical day? (minutes) | Physical activity was measured using the WHO Global Physical Activity Questionnaire (GPAQ)^17^  **Computation of MET–minutes:**   - Walking or cycling MET–minutes/week = 4.0*walking minutes*walking days - Moderate MET–minutes/week = 4.0*moderate–intensity activity minutes*moderate–intensity - Vigorous MET–minutes/week at work = 8.0*vigorous–intensity activity minutes*vigorous–intensity - Total MET–minutes/week = sum of total MET–minutes/week of activity computed for each setting   0: Physically active (total Physical Activity MET minutes per week is ≥ 600) 1: Physically inactive (total Physical Activity MET minutes per week is < 600) |
| **Metabolic risk factors** | | |
| Body mass index | **Q2506**. Measured height in centimetres  **Q2507**. Measured weight in kilograms | Asian–Pacific criteria^18^  0: No overweight or obesity (≤ 23 kg/m^2^)  1: Overweight/obesity (> 23 kg/m^2^) |
| Blood pressure | Second and third measurement of respondent’s blood pressure  **Q2502**. Systolic  **Q2502**. Diastolic  **Q2503**. Systolic  **Q2503.** Diastolic | NCEP criteria^12^  0: Normal (mean systolic <130 *and* mean diastolic <85 mmHg)  1: Elevated (mean systolic ≥130 *or* mean diastolic ≥85 mmHg) |
| Hemoglobin A1C | **Q2546**. HbA1c (%) | WHO^19^  0: Normal (4–6.4%)  1: Elevated (≥ 6.5%)  Missing if < 4% (potential machine error) |
| Total cholesterol | **Q2556.** Total Cholesterol (xxx.xx) mg/dL | NCEP criteria^12^  0: Normal (< 240mg/dL)  1: Elevated (≥ 240mg/dL) |
| Total triglycerides | **Q2560**. Triglycerides (xxx.xx) mg/dL | NCEP criteria^12^  0: Normal (< 150 mg/dL)  1: Elevated (≥150 mg/dL) |
| **Sociodemographic variables** | | |
| Gender | **Q1004**. Record sex of the respondent | 0: Women  1: Men |
| Age groups | **Q1006.** How old are you now? | 0: 18–49  1: 50–59  2: 60–69  3: 70+ |
| Residence area | **Q0104**. Setting (in household questionnaire) | 0: Urban  1: Rural |
| Marital status | **Q1007**. What is your current marital status? | 0: Currently married  1: Never married  2: Divorced/widowed |
| Education level | **Q1011**. What is the highest level of education that you have completed? | 0: At least high school  1: Completed secondary school  2: Completed primary school  3: Incomplete primary  4: Never schooling |
| Household economic group | Wealth index was created based on housing conditions (**Q0501– Q0557**) and assets indicator (**Q0701–Q0722**).  Housing conditions and asset indicators were selected as preliminary variables based on previous guideline^20^, such as residential’s ownership status, materials of walls, floors, and roofs, cooking fuel type, shared toilet use, toilet location, drinking water source, handwashing facilities, and asset ownership (e.g., TV, motorcycle, car, electricity). The Kaiser–Meyer–Olkin (KMO) test was applied to assess the suitability of the selected variables for Principal Component Analysis (PCA). The wealth index scores from the first principal component were ranked in ascending order and divided into five equal groups (quintiles), from Q1 (poorest) to Q5 (wealthiest) | 0: Q5 (wealthiest)  1: Q4  2: Q3  3: Q2  4: Q1 (poorest) |
