# Supplementary material for: Identifying Subgroups At-Risk for Noncommunicable Diseases in Cambodia: A Latent Class Analysis of Behavioral and Metabolic Risk Factor Patterns
Source: J Epidemiol Glob Health. 2025 Oct 13;15(1):119. doi: 10.1007/s44197-025-00464-0 (PMC12518195; doi:10.1007/s44197-025-00464-0)
Supplement: Supplementary file 2 — Supplementary file2 (DOCX 14 KB) [file 44197_2025_464_MOESM2_ESM.docx]

**Additional Table A2**. Total respondents and missing data

|  | **n** | **%** |
| --- | --- | --- |
| **All Cambodia WHS+ respondents** | **6074** |  |
| Excluded respondents due to incomplete or unsuccessful interview  Excluded respondents due to duplicate interview | 797  2 |  |
| **Full sample for analysis** | **5275** | **100** |
| **N without missing value** | 4005 | 75.9 |
| **Variables with missing value**: |  |  |
| Tobacco use | 127 | 2.4 |
| Alcohol consumption | 21 | 0.4 |
| Fruit and vegetables intake | 86 | 1.6 |
| Body Mass Index | 8 | 0.2 |
| Blood pressure | 285 | 5.4 |
| Hemoglobin A1C | 913 | 17.3 |
| Total cholesterol | 318 | 6.0 |
| Total triglycerides | 320 | 6.1 |
| Household economic group | 193 | 3.7 |

Missing data was handled in two ways. FIML was employed for the LCA, while MICE were used to address missing data in the household economic group variable in the multinomial logistic regression.
