# Supplementary material for: Identifying Subgroups At-Risk for Noncommunicable Diseases in Cambodia: A Latent Class Analysis of Behavioral and Metabolic Risk Factor Patterns
Source: J Epidemiol Glob Health. 2025 Oct 13;15(1):119. doi: 10.1007/s44197-025-00464-0 (PMC12518195; doi:10.1007/s44197-025-00464-0)
Supplement: Supplementary file 3 — Supplementary file3 (DOCX 22 KB) [file 44197_2025_464_MOESM3_ESM.docx]

**Additional Table A3.** Full multinomial logistic regression of sociodemographic characteristics predicting latent class membership in relation to Class 1 (alcohol user with lower metabolic risk) (n=5275).

| **Sociodemographic variables** | | | |  | |  | **Class 2** | | | |  | |  | | **Class 3** | | |
| --- | --- | --- | --- | --- | --- | --- | --- | --- | --- | --- | --- | --- | --- | --- | --- | --- | --- |
|  |  |  |  |  | |  | ***Substance user with compounding unhealthy behaviors*** | | | |  | |  | | ***Alcohol user with higher***   ***metabolic risk*** | | |
|  |  |  |  | RRR | (95% CI) | | | *SE* | *t-value* | *p*–value | RRR | (95% CI) | | *SE* | | *t-value* | *p*–value |
|  |  | | **Gender (ref women)** | | | | | | | | | | |  | |  |  |
| Men | |  | | 14.33 | (9.86–20.8) | | | 2.73 | 13.9 | <0.001 | 1.87 | (1.50–2.33) | | 0.212 | | 5.52 | <0.001 |
| **Age group** (ref 18–39 years) | | | | |  | | |  |  |  |  |  | |  | |  |  |
| 40-49 | | | | 1.25 | (0.76– 2.06) | | | 0.319 | 0.88 | 0.379 | 1.90 | (1.42– 2.54) | | 0.280 | | 4.36 | <0.001 |
| 50–59 | | | | 2.67 | (1.65–4.32) | | | 0.656 | 4.01 | <0.001 | 3.13 | (2.28–4.40) | | 0.510 | | 7.01 | <0.001 |
| 60–69 | | | | 1.43 | (0.80–2.59) | | | 0.433 | 1.20 | 0.228 | 2.51 | (1.71–3.68) | | 0.492 | | 4.69 | <0.001 |
| 70+ | |  | | 1.78 | (0.86–3.71) | | | 0.667 | 1.55 | 0.122 | 2.11 | (1.31–3.39) | | 0.512 | | 3.06 | 0.002 |
| **Residence area** (ref urban) | | | | |  | | |  |  |  |  |  | |  | |  |  |
| Rural | |  | | 1.07 | (0.74–1.55) | | | 0.202 | 0.36 | 0.717 | 0.62 | (0.50–0.77) | | 0.068 | | -4.34 | <0.001 |
| **Marital status** (ref currently married) | | | | |  | | |  |  |  |  |  | |  | |  |  |
| Never married | | | | 0.48 | (0.25–0.86) | | | 0.168 | -2.08 | 0.037 | 0.51 | (0.35–0.78) | | 0.099 | | -3.46 | 0.001 |
| Divorced/Widowed | | |  | 1.14 | (0.65–2.04) | | | 0.337 | 0.47 | 0.636 | 1.10 | (0.79–1.53) | | 0.187 | | 0.55 | 0.583 |
| **Education level** (ref at least high school) | | | | | | | |  |  |  |  |  | |  | |  |  |
| Completed secondary | | | | 1.50 | (0.69–3.28) | | | 0.598 | 1.02 | 0.308 | 0.94 | (0.63–1.39) | | 0.189 | | 0.743 | 0.743 |
| Completed primary | | | | 2.26 | (1.08–4.74) | | | 0.854 | 2.16 | 0.031 | 1.48 | (1.02–2.15) | | 0.280 | | 1.02 | 0.038 |
| Incomplete primary | | | | 2.76 | (1.31–5.76) | | | 1.04 | 2.69 | 0.007 | 1.36 | (0.92–1.99) | | 0.264 | | 0.928 | 0.115 |
| Never schooling | | | | 2.81 | (1.31–6.07) | | | 1.10 | 2.64 | 0.008 | 1.04 | (0.67–1.60) | | 0.229 | | 0.18 | 0.859 |
| **Household economic group** (ref Q5 wealthiest) | | | | | | | |  |  |  |  |  | |  | |  |  |
| Q4 | | | | 1.08 | (0.60–1-95) | | | 0.328 | 0.27 | 0.789 | 0.74 | (0.53–1.01) | | 0.120 | | -1.88 | 0.060 |
| Q3 | | | | 1.43 | (0.80–2.55) | | | 0.422 | 1.23 | 0.222 | 0.71 | (0.51–0.97) | | 0.115 | | -2.12 | 0.034 |
| Q2 | | | | 1.31 | (0.72–2.37) | | | 0.398 | 0.89 | 0.375 | 0.53 | (0.38–0.74) | | 0.091 | | -3.70 | <0.001 |
| Q1 poorest | |  | | 2.15 | (1.21–3.81) | | | 0.628 | 2.64 | 0.008 | 0.62 | (0.44–0.88) | | 0.110 | | -2.69 | 0.007 |

CI: confidence interval; Ref: reference group; RRR: relative risk ratio

Reference category for risk factor classes was Class 1 ‘Alcohol user with lower metabolic risk’.
