# Supplementary material for: Identifying Subgroups At-Risk for Noncommunicable Diseases in Cambodia: A Latent Class Analysis of Behavioral and Metabolic Risk Factor Patterns
Source: J Epidemiol Glob Health. 2025 Oct 13;15(1):119. doi: 10.1007/s44197-025-00464-0 (PMC12518195; doi:10.1007/s44197-025-00464-0)
Supplement: Supplementary file 4 — Supplementary file4 (DOCX 20 KB) [file 44197_2025_464_MOESM4_ESM.docx]

**Additional Table A4.** Crude relative risk ratio of sociodemographic characteristics and latent class membership (weighted).

| **Sociodemographic variables** | | **Class 2** | | | **Class 3** | | |
| --- | --- | --- | --- | --- | --- | --- | --- |
|  |  | ***Substance user with compounding unhealthy behaviors*** | | | ***Alcohol user with higher*** ***metabolic risk*** | | |
|  |  | _c_RRR | (95% CI) | *p*–value | _c_RRR | (95% CI) | *p*–value |
| **Gender (ref women)** | | | | | | |  |
| Men |  | 12.40 | (8.67–17.7) | <0.001 | 1.79 | (1.46–2.20) | <0.001 |
| **Age group** (ref 18–39 years) | |  |  |  |  |  |  |
| 40–49 | | 1.86 | (1.21–2.88) | 0.005 | 2.51 | (1-91–3.32) | <0.001 |
| 50–59 | | 3.38 | (2.16–5.26) | <0.001 | 3.80 | (2.80–5.15) | <0.001 |
| 60–69 | | 2.37 | (1.44–3.91) | 0.001 | 3.44 | (2.45–4.82) | <0.001 |
| 70+ |  | 2.67 | (1.46–4.88) | 0.001 | 2.77 | (1.80–4.24) | <0.001 |
| **Residence area** (ref urban) | |  |  |  |  |  |  |
| Rural |  | 1.18 | (0.84–1.66) | 0.355 | 0.57 | (0.47–0.70) | <0.001 |
| **Marital status** (ref currently married) | | |  |  |  |  |  |
| Never married | | 0.49 | (0.27–0.90) | 0.022 | 0.39 | (0.27–0.56) | <0.001 |
| Divorced/Widowed |  | 0.84 | (0.51–1.37) | 0.484 | 1.28 | (0.93–1.76) | 0.129 |
| **Education level** (ref at least high school) | | |  |  |  |  |  |
| Completed secondary | | 1.35 | (0.63–2.90) | 0.437 | 0.85 | (0.59–1.23) | 0.390 |
| Completed primary | | 2.34 | (1.17–4.66) | 0.016 | 1.40 | (1.01–1.96) | 0.047 |
| Incomplete primary | | 3.42 | (1.74–6.69) | <0.001 | 1.45 | (1.04–2.01) | 0.026 |
| Never schooling | | 2.75 | (1.36–5.55) | 0.005 | 1.11 | (0.76–1.60) | 0.590 |
| **Household economic group** (ref Q5 wealthiest) | | |  |  |  |  |  |
| Q4 | | 1.16 | (0.66–2.01) | 0.607 | 0.74 | (0.55–1.00) | 0.050 |
| Q3 | | 1.41 | (0.81–2.43) | 0.223 | 0.64 | (0.48–0.87) | <0.001 |
| Q2 | | 1.37 | (0.79–2.37) | 0.256 | 0.47 | (0.35–0.65) | 0.004 |
| Q1 poorest |  | 2.58 | (1.53–4.35) | <0.001 | 0.61 | (0.44–0.85) | 0.050 |

CI: confidence interval; Ref: reference group; _c_RRR: crude relative risk ratio

Reference category for risk factor classes was Class 1 ‘Alcohol user with lower metabolic risk’.
