# Supplementary material for: Identifying Subgroups At-Risk for Noncommunicable Diseases in Cambodia: A Latent Class Analysis of Behavioral and Metabolic Risk Factor Patterns
Source: J Epidemiol Glob Health. 2025 Oct 13;15(1):119. doi: 10.1007/s44197-025-00464-0 (PMC12518195; doi:10.1007/s44197-025-00464-0)
Supplement: Supplementary file 5 — Supplementary file5 (DOCX 14 KB) [file 44197_2025_464_MOESM5_ESM.docx]

**Additional Table A5**. Goodness of fit for latent class models, complete cases only (n = 3515)

| Model | LL | df | AIC | BIC | Entropy |
| --- | --- | --- | --- | --- | --- |
| 1 | -39873618.12 | 9 | 79747254.23 | 79747309.71 | NA |
| 2 | -38814922.52 | 19 | 77629883.04 | 77630000.18 | 0.889 |
| **3** | **-38588818.41** | **29** | **77177694.82** | **77177873.59** | **0.859** |
| 4 | -38553344.44 | 37 | 77106762.87 | 77106990.97 | 0.833 |
| 5 | -38473836.71 | 48 | 76947769.41 | 76948065.32 | 0.799 |
| 6 | -38426336.55 | 57 | 76852787.11 | 76853138.5 | 0.785 |

LL: Log likelihood; df: Degrees of Freedom; AIC: Akaike Information Criterion; BIC: Bayesian Information Criterion; CAIC; Consistent Akaike Information Criterion.
