# Supplementary material for: Identifying Subgroups At-Risk for Noncommunicable Diseases in Cambodia: A Latent Class Analysis of Behavioral and Metabolic Risk Factor Patterns
Source: J Epidemiol Glob Health. 2025 Oct 13;15(1):119. doi: 10.1007/s44197-025-00464-0 (PMC12518195; doi:10.1007/s44197-025-00464-0)
Supplement: Supplementary file 6 — Supplementary file6 (DOCX 15 KB) [file 44197_2025_464_MOESM6_ESM.docx]

**Additional Table A6.** Prevalence of latent classes and conditional item probabilities, complete cases only (n = 3515)

| **Indicators** | **Class 1**  **37.7%** | **Class 2**  **15.2%** | **Class 3**  **47.1%** |
| --- | --- | --- | --- |
| Current tobacco user | 0.10 | 0.60 | 0.12 |
| Current alcohol consumer | 0.33 | 0.79 | 0.45 |
| Inadequate fruit and vegetable diet | 0.52 | 0.66 | 0.53 |
| Physically inactive | 0.36 | 0.33 | 0.34 |
| Overweight, including obesity | 0.29 | 0.27 | 0.79 |
| Elevated blood pressure | 0.06 | 0.43 | 0.54 |
| Elevated HbA1c | 0.04 | 0.004 | 0.23 |
| Elevated total cholesterol | 0.02 | 0.03 | 0.24 |
| Elevated triglycerides | 0.12 | 0.38 | 0.72 |
