# Supplementary material for: Identifying Subgroups At-Risk for Noncommunicable Diseases in Cambodia: A Latent Class Analysis of Behavioral and Metabolic Risk Factor Patterns
Source: J Epidemiol Glob Health. 2025 Oct 13;15(1):119. doi: 10.1007/s44197-025-00464-0 (PMC12518195; doi:10.1007/s44197-025-00464-0)
Supplement: Supplementary file 7 — Supplementary file7 (DOCX 20 KB) [file 44197_2025_464_MOESM7_ESM.docx]

**Additional Table A7**. Weighted multinomial logistic regression of sociodemographic characteristics predicting latent class membership in relation to Class 2 (n=5275).

| **Sociodemographic variables** | | **Class 1** | | | **Class 3** | | |
| --- | --- | --- | --- | --- | --- | --- | --- |
|  |  | ***Alcohol user with lower metabolic risk*** | | | ***Alcohol user with higher metabolic risk*** | | |
|  |  | RRR | (95% CI) | *p*–value | RRR | (95% CI) | *p*–value |
| **Gender (ref women)** | | | | | | |  |
| Men |  | 0.07 | (0.04–0.10) | <0.001 | 0.13 | (0.09–0.19) | <0.001 |
| **Age group** (ref 18–39 years) | |  |  |  |  |  |  |
| 40–49 | | 0.80 | (0.49–1.32) | 0.381 | 1.52 | (0.93–2.48) | 0.092 |
| 50–59 | | 0.37 | (0.23–0.60) | <0.001 | 1.17 | (0.74–1.84) | 0.495 |
| 60–69 | | 0.70 | (0.39–1.26) | 0.228 | 1.75 | (1.03–2.95) | 0.037 |
| 70+ | | 0.56 | (0.27–1.17) | 0.122 | 1.18 | (0.61–2.29) | 0.619 |
| **Residence area** (ref urban) | |  |  |  |  |  |  |
| Rural |  | 0.93 | (0.65–1.35) | 0.719 | 0.58 | (0.41–0.82) | 0.002 |
| **Marital status** (ref currently married) | | |  |  |  |  |  |
| Never married | | 2.05 | (1.04–4.01) | 0.037 | 1.05 | (0.51–2.14) | 0.899 |
| Divorced/Widowed |  | 0.87 | (0.49–1.54) | 0.636 | 0.96 | (0.57–1.60) | 0.864 |
| **Education level** (ref at least high school) | | |  |  |  |  |  |
| Completed secondary | | 0.67 | (0.30–1.45) | 0.307 | 0.62 | (0.28–1.35) | 0.230 |
| Completed primary | | 0.44 | (0.21–0.93) | 0.031 | 0.65 | (0.32–1.35) | 0.251 |
| Incomplete primary | | 0.36 | (0.17–0.76) | 0.007 | 0.49 | (0.24–1.01) | 0.054 |
| Never schooling | | 0.35 | (0.16–0.77) | 0.008 | 0.37 | (0.17–0.78) | 0.009 |
| **Household economic group** (ref Q5 wealthiest) | | |  |  |  |  |  |
| Q4 | | 0.93 | (0.51–1.68) | 0.807 | 0.69 | (0.39–1.20) | 0.191 |
| Q3 | | 0.70 | (0.39–1.25) | 0.224 | 0.50 | (0.29–0.86) | 0.002 |
| Q2 | | 0.77 | (0.42–1.40) | 0.385 | 0.40 | (0.23–0.72) | 0.013 |
| Q1 poorest |  | 0.47 | (0.26–0.82) | 0.008 | 0.29 | (0.17–0.50) | <0.001 |

CI: confidence interval; Ref: reference group; RRR: relative risk ratio

Reference category for risk factor classes was Class 2 “Substance user with compounding unhealthy behaviors.”
