# Supplementary material for: Identifying Subgroups At-Risk for Noncommunicable Diseases in Cambodia: A Latent Class Analysis of Behavioral and Metabolic Risk Factor Patterns
Source: J Epidemiol Glob Health. 2025 Oct 13;15(1):119. doi: 10.1007/s44197-025-00464-0 (PMC12518195; doi:10.1007/s44197-025-00464-0)
Supplement: Supplementary file 8 — Supplementary file8 (DOCX 15 KB) [file 44197_2025_464_MOESM8_ESM.docx]

**Additional Table A8.** Prevalence of latent classes and conditional item probabilities with sampling weights for 6-class model.

| **Indicators** | **Class 1**  **(9.0%)** | **Class 2**  **(38.3%)** | **Class 3**  **(14.6%)** | **Class 4**  **(26.8%)** | **Class 5**  **(9.0%)** | **Class 6**  **(2.0%)** |
| --- | --- | --- | --- | --- | --- | --- |
| Current tobacco user | 1.00 | 0.02 | 0.13 | 0.17 | 0.10 | 0.59 |
| Current alcohol consumer | 0.52 | 0.33 | 0.005 | 0.99 | 0.21 | 0.42 |
| Inadequate fruit and vegetable diet | 0.64 | 0.54 | 0.49 | 0.53 | 0.54 | 0.80 |
| Physically inactive | 0.31 | 0.33 | 0.31 | 0.24 | 0.34 | 0.43 |
| Overweight, including obesity | 0.10 | 0.34 | 0.55 | 0.78 | 0.90 | 0.41 |
| Elevated blood pressure | 0.31 | 0.13 | 0.23 | 0.49 | 0.62 | 0.48 |
| Elevated HbA1c | 1.31*10^-8^ | 0.03 | 0.14 | 0.06 | 0.37 | 0.18 |
| Elevated total cholesterol | 0.004 | 0.02 | 0.31 | 0.16 | 0.25 | 0.14 |
| Elevated triglycerides | 0.12 | 0.10 | 0.79 | 0.69 | 0.68 | 0.62 |
